# Vaccination in infected children: a qualitative study of clinical decision-making

**DOI:** 10.1017/S1463423625100790

**Published:** 2026-01-20

**Authors:** Ayse Esra Tapci, Edanur Acarel, Izzet Fidanci, Medine Aysin Tasar

**Affiliations:** 1 Department of Pediatrics, University of Health Sciences Ankara Training and Education Hospital, Ankara, Türkiye; 2 Department of Family Medicine, Hacettepe University, Faculty of Medicine, Ankara, Türkiye; 3 Department of Pediatric Emergency Medicine, University of Health Sciences Ankara Training and Education Hospital, Ankara, Türkiye

**Keywords:** clinical decision-making, family practice, paediatrics, qualitative research, vaccination, vaccine hesitancy

## Abstract

**Aim:**

This study aimed at investigating the clinical, individual, and systemic factors influencing paediatricians’ and family physicians’ clinical decision-making process in the vaccination of children during infection from the physician’s perspective.

**Methods:**

A qualitative study through semi-structured in-depth interviews was conducted among 10 paediatricians and 10 family physicians working in Ankara, Türkiye. The audio-recorded interviews were translated into written texts, and the obtained data was analysed using the thematic analysis method proposed by Braun and Clarke.

**Results:**

Four main themes were identified in of thematic analysis: (I) Impact of clinical conditions on vaccination decisions, (II) attitudes of families and their communication processes with physicians, (III) impact of practice settings and institutional factors, and (IV) vaccine postponement and compensation approaches. It was observed that the decision-making processes of the paediatricians were mainly based on the clinical evaluation criteria, while family physicians considered the expectations of the families and institutional conditions. Also, the importance of establishing effective communication with vaccine-hesitant families has been emphasized by both groups of physicians.

**Conclusion:**

In the immunization of infected children, decision-making is shaped in addition to medical facts in relation to the parental attitude, organizational factors within health institutions, and personal experiences of medical staff. Decisions of paediatricians are largely grounded in medical facts, whereas family practitioners assess that social and organizational factors are of higher importance. Improved adherence to medical guidelines and communication competencies of medical professionals can contribute towards medical practice consistency.

## Introduction

Vaccination is recognized as one of the most effective public health interventions for preventing childhood infections. The World Health Organization reports that millions of child deaths are prevented by vaccines every year (WHO, 2025). The contribution of vaccination to global health, especially in low- and middle-income countries, is considered one of the most important achievements of modern medicine; it prevents more than four million deaths each year (WHO, 2019; Adeyanju *et al*., 2022). Vaccination services are among the most effective and cost-effective methods for controlling infectious diseases and reducing mortality and morbidity in children, especially when full compliance with the vaccination schedule is achieved (Dannetun *et al*., 2004; Lee *et al*., 2005). Currently, one of the main reasons for inadequate vaccination rates is missed vaccination opportunities. Missed vaccination opportunity is the situation in which timely vaccines cannot be administered for various reasons without any contraindication despite the individual’s presentation to a healthcare institution (Arvas, 2009). Various clinical conditions, parental concerns, and different approaches of healthcare professionals have led to the postponement of vaccinations. Although clinical guidelines make it clear that mild infections are not a barrier to vaccination, decision-making in practice is influenced by symptom severity, parental attitude, and practice environment (Siegrist, 2013; Kroger *et a*l., 2017). Parental hesitancy about vaccine safety can further complicate this process altogether (Smith *et al*., 2011).

In this case, this research proposes to explore factors that affect decision-making in vaccine administration among children suffering from infections from the perspective of physicians.

## Methods

### Research design

In this proposed study, the researcher utilized an interpretive qualitative approach that aims for an intensive analysis of children face decisions to vaccinate those who contracted infections. In this case, this proposed study utilized an interpretive approach to qualitative analysis that has its roots in thematic analysis. In reporting this proposed qualitative study, this work followed the Consolidated Criteria for Reporting Qualitative Research (COREQ), the qualitative study guidelines for reporting (Tong *et al*., 2007), and the standards of the Equator Network (2025)

### Participants and sampling

In this current study, purposive sampling is used for selecting samples. In this case, this research ended up with a total of 20 participants, including 10 paediatricians working in the Department of Pediatrics of Ankara training and research hospital in Ankara, Türkiye and another 10 family physicians practicing in the Family Healthcare Centers (consisting of 8 Family Health Care Centers, 1 University Hospital, and 1 District State Hospital). Participants were selected based on their knowledge and experience in sharing their experiences about health services. The sample was chosen from physicians with at least one year of experience, providing active immunization services, and working with infected children. The sociodemographic characteristics of the participants are presented in Table 1.

**Table 1.** Socio-demographic characteristics of the participants

Participant*	Age	Gender	Marital status	Parental status	Employed institution
**P1**	29	Woman	Married	No	Training and Research Hospital
**P2**	31	Woman	Married	Yes	Training and Research Hospital
**P3**	42	Woman	Married	Yes	Training and Research Hospital
**P4**	54	Woman	Married	Yes	Training and Research Hospital
**P5**	34	Male	Married	Yes	Training and Research Hospital
**P6**	40	Woman	Married	Yes	Training and Research Hospital
**P7**	35	Male	Married	Yes	Training and Research Hospital
**P8**	34	Woman	Married	No	Training and Research Hospital
**P9**	38	Woman	Married	Yes	Training and Research Hospital
**P10**	35	Male	Married	Yes	Training and Research Hospital
**FP1**	42	Woman	Married	Yes	University Hospital
**FP2**	52	Male	Married	Yes	Family Healthcare Center
**FP3**	47	Woman	Single	No	Family Healthcare Center
**FP4**	29	Woman	Single	No	Family Healthcare Center
**FP5**	37	Male	Married	Yes	Family Healthcare Center
**FP6**	35	Male	Married	No	Family Healthcare Center
**FP7**	30	Male	Single	No	District State Hospital
**FP8**	31	Woman	Married	No	Family Healthcare Center
**FP9**	27	Male	Married	No	Family Healthcare Center
**FP10**	30	Woman	Married	Yes	Family Healthcare Center

*P: Paediatrician, FP: Family Physician.

A purposive sampling method was adopted for recruiting physicians with direct experiences in vaccination practices among children presenting with infections. This approach allowed us to intentionally select participants who could provide rich, relevant, and experience-based information in line with the study aim. As qualitative research deals with depth of understanding rather than representativeness, a purposive sampling method would ensure that data were obtained from those who could meaningfully describe the decision-making process in clinical practice.

### Data collection process

The data collection process took place in March 2025. One-to-one, semi-structured in-depth interviews were conducted with all participants. The interviews were conducted using a pre-prepared form with open-ended questions. Each interview lasted for an average of 5 minutes (± 1 min 36 seconds). The interviews were audio-recorded with permission from the participants, transcribed verbatim, and converted into written text. The audio recordings were securely destroyed within 24 h of transcription, in line with the ethical principles.

### Interview guide and process

Based on the literature review and expert opinions, the interview form was prepared as semi-structured, considering the main themes such as vaccination decision, communication with families, vaccine postponement, and compensation strategies in children with infections. After a pilot test, which was conducted to test the interview guide (Appendix 1), the content and expression were revised and finalized. One of the researchers carried out the process of interviewing, while the other observed and recorded notes. In this aspect, the process aims to improve the quality of the data and assess the interview process as well.

### Interviewer characteristics

All interviews were performed by a family practice physician trained in qualitative research and in semi-structured interviewing techniques. The interviewer possessed a doctorate in her field and had never met any of the participants in this or any other professional capacity. Before engaging in each interview, the participant had been informed of the nature of the research and that the interview would be voluntary and that individual responses would be private. No other personal characteristics besides sex were acknowledged in preparation for the interviews to prevent affecting the neutral interviewing attitude.

### Data analysis

Braun and Clarke’s approach to thematic analysis was utilized on the data collected (Braun and Clarke, 2006). In the initial stages, researchers studied all the transcripts multiple times to gain acquaintance with the data. Subsequently, coding by two different researchers was carried out before a consensus agreement on the themes was attained. Coding was further grouped into major and minor themes based on similarities of the content. The themes were associated with the research questions and reported as meaningful. To avoid giving a misleading quantitative impression, numerical counts and percentages were not used when reporting the findings. Instead, interpretive frequency labels were applied as recommended for qualitative research. In this study, ‘a few’ referred to 1–2 participants, ‘several’ to 3–5 participants, ‘some’ to 6–10 participants, ‘many’ to 11–15 participants, and ‘most’ to 16–20 participants.

### Ethical principles

The study was carried out with the approval of the Scientific Research Ethics Committee of University of Health Sciences, Ankara Training and Research Hospital (Meeting Date: 05.02.2025; Decision No: E-25-391). All participants provided written informed consent. Participants’ identities were kept confidential; audio recordings of interviews were used only for research purposes and securely destroyed within 24 hours after transcription was completed. Regarding reporting of findings, participants were coded as Family Physician (FP) and paediatrician (P) according to their professional identities.

### Reliability, validity, and reporting standards

Iterative steps of coding and categorization were carried out for thematic analysis to increase reliability. Direct quotes from the participants were used in support of the findings’ validity. The reliability of the study has been ensured by cross-checking the coding process. Coding was performed independently, and variation in interpretation was settled by mutual agreement. Internal validity has been ensured by reporting the research process and the steps of thematic analysis in detail. The current study has been designed and reported in correspondence with the COREQ checklist, which has been developed within the EQUATOR Network for making qualitative research more transparent and scientifically reported (Tong *et al*., 2007; Equator Network, 2025). Participants were selected through a purposeful sampling strategy aimed at acquiring a diverse sample that could represent sociodemographic variability with respect to age, professional experience, and the level of education. The interviews were carried out in compliance with ethical considerations regarding the assurance of confidentiality and comfort for the participants.

### Researcher characteristics and reflexivity

These interviews were conducted by an experienced research team with deep knowledge in qualitative research methodologies and public health. All interviews were performed on an individual basis by a female physician. A researcher who conducted the interviews had no prior professional or personal contact with participants and maintained a professional attitude, being empathetic but non-judgemental during the interview. The principle of reflexivity has been observed during the whole process of the study, and the influence of subjective biases and assumptions of researchers on data analysis has been minimized. In this context, the analysis process was carried out within a permanently present awareness of the researcher in accordance with methodological rigor

## Results

The mean age of the participants was 36.6 ± 7.6 years, ranging from 27 to 54 years. The mean age of paediatricians and family physicians was 37.2 ± 7.1 (min: 27; max: 52) and 36.0 ± 8.5 (min: 29; max: 54) years, respectively. Other sociodemographic characteristics of the participants are presented in Table 1.

One of the paediatricians (P7) postponed the vaccination of his child due to an upper respiratory tract infection, while another (P9) postponed the vaccination of his child due to a diagnosis of neutropenia. Among the participants who had children from family physicians, only one family physician (FP1) postponed the vaccination of his child due to premature birth.

Data obtained in this study were analysed to reveal clinical, individual, and systemic factors in the processes of making vaccination decisions in children during infection. Thematic analysis was used; thus, findings were structured into four overall themes:

- Clinical conditions affecting the vaccination decision process
- Family attitudes and physician-family communication,
- Implementation practices and institutional processes,
- Vaccine deferral and compensation approaches.

In each theme, the approaches of paediatricians and family physicians were evaluated comparatively, and the findings were supported with direct participant quotes in accordance with qualitative data analysis (Table 2).

**Table 2.** Thematic coding table

Theme	Major category	Minor categories
I. Clinical conditions affecting the vaccination decision process	Vaccination decision in the presence of infection	- Type and severity of infection - Evaluating systemic symptoms - Considering infection duration - Presence of fever - Parent-reported vs. physician-detected fever - Interpreting clinical red flags - Child’s general condition
	Decision-making based on vaccine type	- Assessment by vaccine type - Assessing contraindications - Adjusting timing based on vaccine content - Previous adverse reaction history - Risk–benefit evaluation - Multi-dose vaccine scheduling issues
II. Family attitudes and communication	Communication with the patient	- Clarifying parental misconceptions - Explaining safety during mild infections - Reducing anxiety - Tailoring explanations - Reassurance strategies - Handling vaccine fears
	Sharing the decision with the patient	- Collaborative decision-making - Evaluating parental readiness - Negotiating postponement - Maintaining trust - Communicating alternatives - Balancing parental requests
III. Implementation practices and institutional processes	Application differences	- Institutional workflow variation - Workload differences - Vaccine availability - Documentation workload - Resource constraints
	Application protocols	- Variability in interpreting guidelines - Protocol inconsistencies - Institutional rule pressure - Ambiguous guideline areas - Awareness of updated recommendations
	Performance system	- Appointment limitations - Time pressure - Performance-linked biases - Documentation targets - Administrative delays
IV. Vaccine delay and compensation approaches	Reasons for postponement	- Parental refusal - Physician caution - Fever persistence - Concern about worsening symptoms - Family hesitation - Workload-related postponement
	Compensation strategies	- Individualized catch-up schedules - Adjusting intervals - Minimizing delay impact - Communicating follow-up plans - Tracking missed appointments

Overall, the analysis yielded that paediatricians and family physicians, though grounded on similar clinical principles in assessing vaccination during infection, differed in the importance given to parental concerns, institutional conditions, and communication-related factors in their decision-making processes.

- Clinical conditions affecting the vaccination decision process

Some participants (*n* = 6) stated that the decision to vaccinate children with signs of infection was based on factors such as the child’s general clinical condition, severity of the infection, and type of vaccine to be administered. Vaccination was generally not postponed in children with mild symptoms, whereas a tendency to postpone vaccination was more frequent in children with fever and systemic symptoms.

Analyses related to this theme revealed that all paediatricians considered clinical status as the primary determinant in the decision to vaccinate, whereas some family physicians also considered parental requests as a primary factor in the decision process.

“If there is a slight runny nose, I give the vaccine. But if he is weak, I will postpone it.” (P3)

“We vaccinate if the child’s temperature is below 38.5.” (FP6)

“If there is a high fever, I postpone if possible.” (FP1)

“If the mother is anxious, then sometimes I postpone it.” (P1)

“I can vaccinate a child who is on antibiotics but does not have a fever.” (P2)

- **Family attitudes and communication**

All family physicians participating in the study stated that parents may exhibit a cautious approach to vaccination in children with infections and that this situation brings about a persuasion process. It was observed that family physicians applied different communication strategies to address parents’ concerns.

While all family physicians emphasized that establishing a long-term relationship builds trust in families and provides an advantage in persuasion processes, some of paediatricians stated that they carry out the process of persuading families with scientific explanations and medical guidance.

“If the family says, ‘my child is sick, let’s postpone it’, sometimes I convince them.” (FP1)

“I don’t do it without persuasion. First I reassure them.” (P4)

“Establishing a long-term relationship with the family facilitates the persuasion process.” (FP2)

“I explain, we decide together with the family.” (P2)

“They are afraid of overlapping the vaccination schedule and disease symptoms.” (FP3)

- **Implementation practices and institutional processes**

All family physicians stated that vaccination decisions were based on performance criteria and appointment system. In Family Healthcare Centers, it has been emphasized that the vaccination schedule is based on a specific order and system. For example, some vaccines can only be administered on specific days.

Some of the paediatricians stated that they were able to perform individual clinical assessments based on the Ministry of Health guidelines. These findings suggest that paediatricians are better able to make decisions based on individual clinical assessments in hospitals.

“There is a performance schedule at the Family Healthcare Center, we try not to go beyond it.” (FP2)

“Some of the vaccines can only be given on certain days. BCG vaccine, for example, so we postpone it and move it to the appropriate day.” (FP1)

“If there is no clear statement in the guideline, I make a decision based on my own clinical observation but mostly based on CDC or Ministry guidelines.” (P1)

“If there is a protocol in the institution, I follow it, but mostly I have the chance to make individual evaluations.” (P3)

- **Vaccine delay and compensation approaches**

While all paediatricians based their decision to postpone mostly on clinical findings, some of family physicians stated that they also considered social and communication factors.

The compensation plan after postponement is usually made in accordance with the Ministry’s schedule; however, the physician’s initiative also plays an important role in this process.

“If the child is in intensive care, we postpone it. But if the child is going home, they say it can be done. If the family is uneasy, even if there is a side effect from something else, they will know about the vaccine. That’s why I postpone it.” (FP2)

“We adjust the compensation plan after postponement according to the expiry date.” (FP1)

“Vaccination should not be delayed, especially in the first months of the child’s life, because postponing it in this way disrupts the whole plan. Vaccinations should be done on time as much as possible.” (P7)

“When planning catch-up vaccinations, we often decide together with the family, some families are afraid of the risk of getting a second disease.” (P1)

## Discussion

This study analysed the vaccination decision process in infected children based on the experiences of family physicians and paediatricians, and revealed the individual, institutional, and communicative factors that influence the decision process. The findings show that the decision to vaccinate an infected child is shaped not only by clinical guidelines, but also by the professional experience of the physician, the attitude of the parents, and the functioning of the institution. This suggests that the decision process should be considered not only in a biomedical context but also in a sociocultural context.

In this study, the clinical, individual, and systemic factors affecting the vaccination decision process in children with infections were examined. The findings were grouped into four main themes: (I) clinical conditions affecting the vaccination decision process, (II) family attitudes and communication, (III) implementation practices and institutional processes, and (IV) vaccine postponement and compensation approaches. This section includes an evaluation of the findings based on the existing literature.

- Clinical conditions affecting the vaccination decision process

In our study, most participants reported that they did not delay vaccination in children with mild symptoms but tended to delay vaccination in cases presenting with fever and systemic manifestations. Clinicians or other healthcare providers may misperceive certain conditions or circumstances as valid contraindications or precautions to vaccination when in fact these conditions do not prevent vaccination. These misperceptions lead to missed opportunities in the implementation of recommended vaccines (Yaprak *et al.*, 2005; Taşar and Dallar, 2015). Unrealistic contraindications due to acute or chronic diseases are one of the reasons for incomplete vaccination (Siegrist, 2013; Kroger *et al.*, 2017). In a study conducted in Türkiye, missed vaccination opportunities were reported to be 8.5% in children aged 6 months-6 years admitted to the hospital and 20% of missed vaccination opportunities were reported to be due to false contraindications (Taşar and Dallar, 2015). Existing guidelines provide conflicting, and sometimes ambiguous, recommendations on how to deal with such situations. For example, a recommendation by the American Advisory Committee on Immunization Practices (ACIP) states that ‘*vaccination should not be delayed because of the presence of a mild respiratory tract infection or other acute illness with or without fever.*” Although routine physical examinations and procedures (e.g., measurement of body temperature) are not prerequisites for vaccination of apparently healthy individuals, it is recommended that vaccination be postponed if the child suffers from a moderate or severe illness when the parent is questioned by the healthcare provider to determine whether the child is ill (Kroger *et al*., 2017). In the United Kingdom, it is stated that *‘minor illness without fever or systemic illness is not a valid reason to postpone vaccination’*. If a person is acutely unwell, vaccination can be delayed until the person has fully recovered (UK Health Security Agency, 2013). The French Vaccination Guidelines state that, ‘*contrary to popular belief, minor infectious diseases are not a contraindication to vaccination*’ (INPES, 2016). However, fever was not explicitly mentioned in these recommendations. A study conducted in France in 2014 on a nationally selected sample of general practitioners revealed that 94% of the participating general practitioners reported that they postponed vaccination of a child with febrile cold, and fever was found to be the main factor affecting vaccination decisions (Maréchal *et al*., 2018).

In international literature, it is common for clinicians to not fully comply with guidelines for vaccination decisions. For example, Kennedy *et al*. reported that 35% of paediatricians in the United States delayed vaccination in cases of mild illness (Kennedy *et al*., 2005). Similarly, in a qualitative study conducted in Canada, most family physicians stated that they preferred to postpone the vaccination decision to the next visit, particularly in cases of suspected viral infection (Gagneur *et al*., Gagneur *et al*., 2018). In another study conducted with family physicians and paediatricians in France, postponing vaccination in children with infections was perceived as common and risk-free among physicians. Most physicians postpone vaccination for febrile and non-febrile infections. This practice is learned during medical training and endorsed by experts. Most physicians underestimated the risks of delaying vaccination and considered it natural practice without additional justification. Vaccine postponement is often justified by the uncertainty that an infection may reduce vaccination efficacy or increase the risk of complications. In addition, the decision to postpone is also made to facilitate the differentiation of infection symptoms from vaccine side effects and to avoid forensic risks (Gonthier *et al*., 2020).

Although authorities such as the World Health Organization (WHO) and the American Center for Disease Control and Prevention (CDC) state that conditions such as mild fever, upper respiratory tract infections, or diarrhoea do not constitute a contraindication for vaccination (Kroger *et al*., 2017; Adeyanju *et al*.,^2022^; WHO, 2025), in our study, it was observed that some clinicians postponed vaccination despite a mild course of infection. This shows that clinical guidelines are not always decisive in practice and decisions are often shaped by the physician’s personal assessment. Similar results were also found in Siegrist’s evaluations of the immune system; the author stated that the possibility that the immune system may not respond adequately to vaccines during infection led some physicians to act cautiously (Siegrist, 2013).

- Family attitudes and communication

Participants emphasized that parents may be cautious about vaccination in children with infections, and that this brings about a process of persuasion between the physician and the family. Similarly, in a study by Smith *et al*. involving 2921 parents, it was shown that parents’ concerns about vaccine safety can be reduced by effective communication strategies with physicians. In this study, which examined parents’ deliberate postponement behaviours during the vaccination process for their children and the possible causes and consequences of these behaviours, 21.8% of the parents stated that they deliberately postponed their children’s vaccinations; the most common reasons for this postponement included concerns about the safety and efficacy of vaccines (44.8%) and the child being sick (36.1%) (Smith *et al*., 2011). Although alleviating parental concerns was also an important reason for postponing vaccination, it was reported that physicians decided to postpone vaccination to gain the trust of anxious parents who did not want any additional ‘coercive intervention’ to be performed on their children during infection, and that this approach positively affected the doctor-patient relationship. In the same study, it was observed that physicians had difficulty dealing with vaccine hesitancy and therefore preferred to postpone vaccination in the presence of infection to avoid conflict with parents. Fears of vaccination were generally found to be irrational (Gonthier *et al*., 2020). Qualitative findings show that many physicians consider parental attitudes and clinical findings when making decisions. In a study by Smith *et al*., it was shown that parental hesitancy towards vaccination caused a significant decrease in vaccination rates and that these hesitations were frequently based on the belief that ‘vaccination should not be given when the child is sick’ (Smith *et al*., 2010). This shows that physicians may postpone their decisions owing to parental pressure or approval, even though there is no clinical obstacle to vaccination. These findings contradict the national guidelines in Türkiye. Guidelines published by the Ministry of Health clearly state that mild illnesses do not constitute an obstacle to vaccination (Oğuzöncül *et al*., 2021; Republic of Türkiye Ministry of Health, 2025). Despite this, some participants reported delaying vaccination in children with febrile infections due to parental objection or fear of legal liability. This shows that in practice, ‘preventive attitude’ behaviour rather than ‘preventive medicine’, which influences clinical decision-making, comes to the forefront.

- Implementation Practices and Institutional Processes

Family physicians have reported that immunization decisions are linked to performance measures, appointment systems, and days when the vaccine can be administered. Paediatricians, on the other hand, tend to make decisions by strictly adhering to the guidelines. This situation was also emphasized in a study by Salmon *et al*., who stated that institutional policies were effective in the vaccination practices of healthcare professionals (Salmon *et al*., 2008).

The family physicians’ limited time per patient causes them to be unable to establish detailed communication with the family, which paves the way for postponement of vaccination. In a study conducted by the Infectious Diseases Society, primary care physicians should be supported in terms of communication strategies even if they have a high level of knowledge about vaccination (Oğuzöncül *et al*., 2021). The fact that physicians have the knowledge that ‘vaccination is feasible’ does not always result in the implementation of this knowledge. The decisive factor here is the trust relationship and communication skills between physician and patient/parent.

Paediatricians are more self-confident by relying on their expertise while making decisions, whereas family physicians are more cautious about possible complications. This finding overlaps with the findings of a previous study on vaccination decisions in children with chronic diseases (Polat *et al*., 2012; Özbörü Aşkan and Keskindemirci, 2024). Here, it was revealed that non-clinical factors were determinants of physicians’ decisions, despite their knowledge.

These findings suggest that family physicians are subject to more systemic constraints and make decisions based on institutional protocols, while paediatricians have a more flexible decision-making process based on clinical observations and guidelines.

- Vaccine delay and compensation approaches

It was reported in the interviews that the most common reasons for postponing vaccination were serious infection, parental concerns, and social conditions. It is usually possible to compensate the vaccination plan after postponement in accordance with the official vaccination schedule; however, the physician has an important initiative during this process. This finding was also stated in the study by Dombkowski *et al*. They emphasized the importance of timely vaccination and reducing the postponement to a minimum during this period (Legifrance, 2018; Dombkowski *et al*., 2024). In a survey study performed in France, the most important difficulty determined by the interviewed physicians was the complexity of the special appointment arrangement in order to complete the missing vaccination doses. In the statement, it was mentioned that organizational difficulties existed in this process and that active recall due to ethical and legal limitations was not possible for self-employed physicians. The facilitation of the compensation process by the traceability of the vaccines in health records and the presence of automatic reminder systems (short message service – SMS, digital health record) was emphasized (Gonthier *et al*., 2020). In France, adding mandatory vaccinations to the new Health Law was considered as one of the methods used by public authorities with the purpose of increasing the accountability of parents. With this law, the implementation of 11 compulsory vaccinations that must be administered before sending the child to school is regulated (Legifrance, 2018). The development and implementation of ‘standing order’ protocols for vaccinations increased the rates of vaccination in children. Standing order protocols are a series of written instructions prepared in advance, which enable healthcare personnel-nurses or pharmacists-to provide medical interventions to those patients who meet certain clinical criteria without individual assessment and direct instruction by a physician. Standing order practices in vaccination programmes enable healthcare workers to administer vaccines directly and without delay to eligible paediatric or adult patients, thereby increasing vaccination rates and accelerating the completion of missing vaccines (Frew and Lutz, 2017). Similarly, the development of templates and interfaces for electronic health record systems, availability of vaccines in doctors’ offices, and opportunity for immediate vaccination have also been effective in increasing vaccination rates (Réthoré Berthomé and Birault, 2014; Zimmerman *et al*., 2014).

## Implications for clinical practice

This study demonstrated that the decision to vaccinate children with infections does not depend solely on clinical symptoms but is shaped by multiple variables such as parental attitude, institutional conditions, and physician experience. In this context, the recommendations are as follows:

- Standard Implementation Protocols need to be developed on a site-specific basis.
- Physician training should include communication and negotiation skills, and not just knowledge enhancement.
- The applicability of the guidelines should be assessed considering local conditions.
- Information materials and hotspots for parents should also be established.

## Limitations and future research

The limitations of this study include the relatively small sample size and the fact that the data were collected only from Ankara, Türkiye. In future studies, data should be collected with larger samples from family healthcare centres and children’s hospitals in different regions, and the bilateral dimension of the decision-making process should be analysed in greater depth through interviews with parents. Intervention-based studies (e.g., studies evaluating the effectiveness of educational programmes) should be conducted to address this issue.

## Conclusion

This study shows that physicians’ decisions to vaccinate or not vaccinate infected children result from the interaction of clinical observations, parents’ opinions, organizational procedures, and professional experience. Paediatricians are mostly guided by clinical assessment, whereas family doctors more often consider parents’ concern and structural barriers within primary care. In this context, communicating effectively and building trust between physicians and families is central in dealing with hesitation and avoiding postpones not justified by medical reasons. Enhancing guideline clarity, providing physicians with communication skills training, and optimizing organizational procedures may contribute to greater consistency and timeliness of vaccination practice. Further studies should investigate parents’ perspectives and assess the effectiveness of different strategies for improving missed opportunities.

## Supporting information

Tapci et al. supplementary material Tapci et al. supplementary material

## Data Availability

No new data was generated or analysed in support of this research.

## Supplementary material

The supplementary material for this article can be found at https://doi.org/10.1017/S1463423625100790
