# Supplementary material for: Vaccination in infected children: a qualitative study of clinical decision-making
Source: Prim Health Care Res Dev. 2026 Jan 20;27:e10. doi: 10.1017/S1463423625100790 (PMC12817223; doi:10.1017/S1463423625100790)
Supplement: Tapci et al. supplementary material [file S1463423625100790sup001.pdf]

Appendix 1. Semi-Structured Interview Guide

Introduction

- Brief explanation of the study purpose

- Assurance of confidentiality and voluntary participation

- Permission to record the interview

Section 1: Professional Background

1. Could you briefly describe your professional background (years of experience, current workplace, specialty)?

Section 2: Experiences with Vaccination

2. Have you previously been involved in administering vaccinations?

3. Have you experienced any challenges when planning vaccination schedules for children? Can you share an example?

Section 3: Decision-Making in Children with Infection

4. Have you ever postponed a vaccination? Under what circumstances?

5. How do you approach vaccination when a child presents with mild symptoms such as a cold without fever?

6. How do you approach vaccination when a child presents with fever or signs of acute infection?

Section 4: Perceptions about Postponement

7. What are your general thoughts about postponing vaccinations in children with infections?

8. How do you explain your vaccination decision to parents?

Section 5: Parental Concerns and Vaccine Hesitancy

9. How do you manage situations where parents show anxiety or hesitancy toward vaccination?

Section 6: Catch-up Vaccination Practices

10. When a vaccination is postponed, how do you plan the catch-up schedule?

11. Do you usually make this plan together with the family?

Closing

- Asking if the participant would like to add anything else
- Thanking the participant for their time
